# Evaluating the Impact of State Policy Interventions for LGBT Aging: Comparative Case Study of New York and California

**DOI:** 10.1093/geroni/igaa057.2340

**Published:** 2020-12-16

**Authors:** Austin Oswald, Nancy Giunta, Tim Johnston, Sherrill Wayland

**Affiliations:** 1 The Graduate Center, City University of New York, Long Island City, New York, United States; 2 Silberman School of Social Work, Hunter College, New York, New York, United States; 3 sAGE, New York, New York, United States; 4 SAGE, New York, New York, United States

## Abstract

The majority of aging network service providers are unprepared to deliver targeted services to lesbian, gay, bisexual, or transgender (LGBT) older adults. In 2017, California legislature mandated ongoing LGBT sensitivity training for congregate living providers. Services and Advocacy for GLBT Elders (SAGE) developed a specialized training, Creating Inclusive Communities, for congregate living staff to learn the unique needs of LGBT elders. This secondary data analysis compared pre-test knowledge and attitudes of training participants in two states, one mandating LGBT aging sensitivity training (California, N=328) and one without the mandate (New York, N=622). Preliminary results show that prior to receiving training, California participants demonstrate significantly less knowledge of LGBT aging issues compared to New York participants; t(948)=-3.808, p<.001. Attitudinal differences were also demonstrated. These results suggest that laws mandating LGBT sensitivity training may help reach providers with greater training needs. Policy and practice implications will be discussed. Part of a symposium sponsored by Rainbow Research Group Interest Group.

